# Characterisation of anhydro-sialic acid transporters from mucosa-associated bacteria

**DOI:** 10.1099/mic.0.001448

**Published:** 2024-03-15

**Authors:** Yunhan Wu, Andrew Bell, Gavin H. Thomas, David N. Bolam, Frank Sargent, Nathalie Juge, Tracy Palmer, Emmanuele Severi

**Affiliations:** 1 Microbes in Health and Disease, Biosciences Institute, Newcastle University, Framlington Place, Newcastle upon Tyne NE2 4HH, UK; 2 Quadram Institute Bioscience, Gut Microbes and Health Institute Strategic Programme, Rosalind Franklin Road, Norwich Research Park, Norwich NR4 7UQ, UK; 3 Department of Biology and York Biomedical Research Institute (YBRI), Wentworth Way, University of York, York YO10 5DD, UK

**Keywords:** bacteria, microbiome, mucosa, sialic acid, sialidase, transporter

## Abstract

Sialic acid (Sia) transporters are critical to the capacity of host-associated bacteria to utilise Sia for growth and/or cell surface modification. While N-acetyl-neuraminic acid (Neu5Ac)-specific transporters have been studied extensively, little is known on transporters dedicated to anhydro-Sia forms such as 2,7-anhydro-Neu5Ac (2,7-AN) or 2,3-dehydro-2-deoxy-Neu5Ac (Neu5Ac2en). Here, we used a Sia-transport-null strain of *Escherichia coli* to investigate the function of members of anhydro-Sia transporter families previously identified by computational studies. First, we showed that the transporter NanG, from the Glycoside-Pentoside-Hexuronide:cation symporter family, is a specific 2,7-AN transporter, and identified by mutagenesis a crucial functional residue within the putative substrate-binding site. We then demonstrated that NanX transporters, of the Major Facilitator Superfamily, also only transport 2,7-AN and not Neu5Ac2en nor Neu5Ac. Finally, we provided evidence that SiaX transporters, of the Sodium-Solute Symporter superfamily, are promiscuous Neu5Ac/Neu5Ac2en transporters able to acquire either substrate equally well. The characterisation of anhydro-Sia transporters expands our current understanding of prokaryotic Sia metabolism within host-associated microbial communities.

## Introduction

**Fig. 1.**
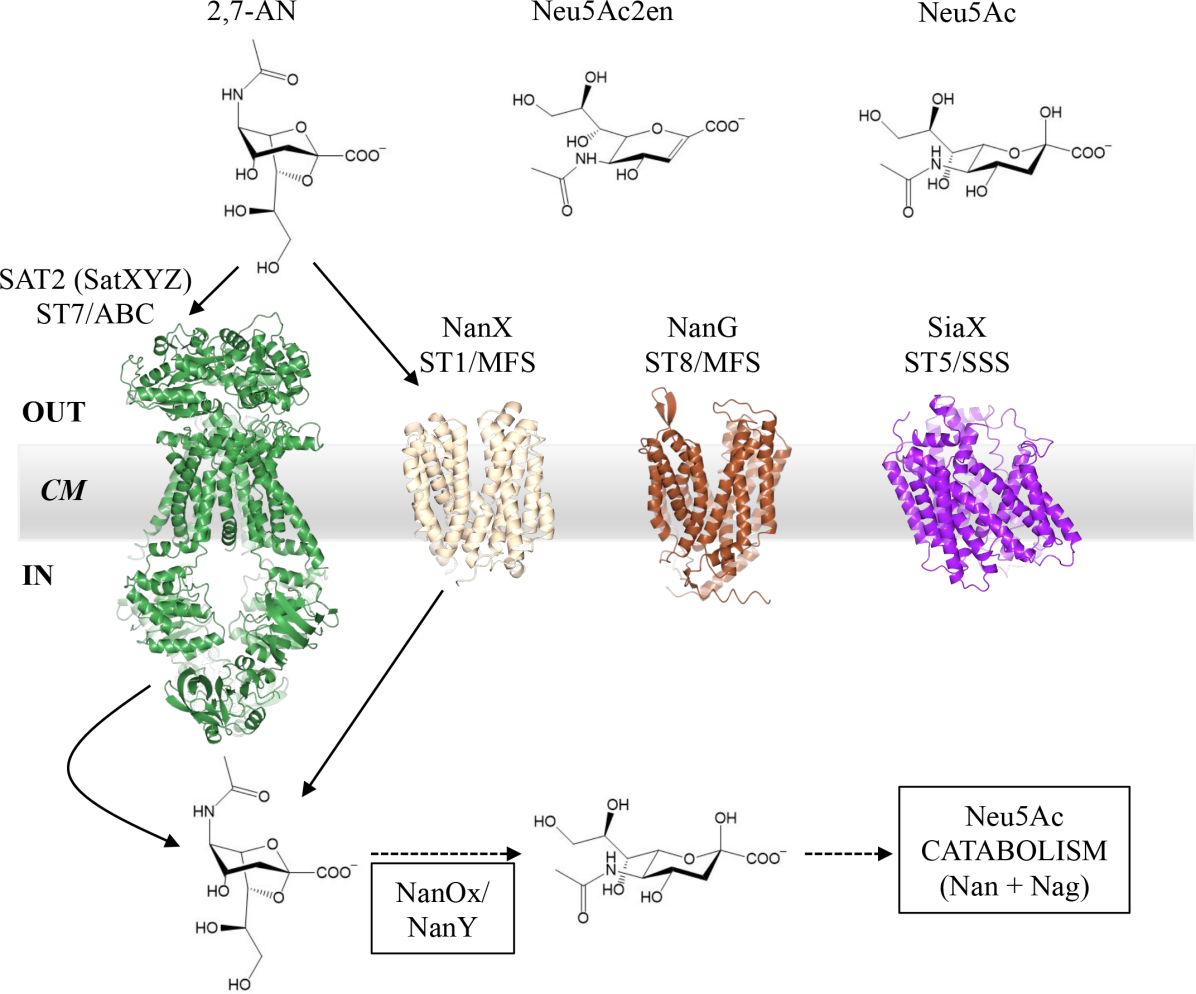
Anhydro-Sia transporters from mucosa-associated bacteria. The four phylogenetically distinct families (ST) of anhydro-Sia transporters are here represented by SAT2 (aka SatXYZ; ST7/ABC) from *R. gnavus* (*Rg*) ATCC 29149, NanX (ST1/MFS) from *E. coli* (*Ec*) BW25113, and NanG (ST8/MFS) and SiaX (ST5/SSS), both from *S. lutrae* DSM 10244. Sia ABC transporters are multidomain complexes comprising an extracytoplasmic solute-binding protein, a dimeric transmembrane domain, and a dimeric cytoplasmic ATPase domain which energises transport by ATP hydrolysis [41]. In contrast, Sia MFS and SSS systems are secondary (i.e. ion gradient-dependent) transporters comprising a single protein domain, made of structural repeats in either case, but with different folds and internal symmetries between repeats [42,43]. While both from the MFS superfamily, ST1 and ST8 transporters belong to distinct families [17] which function by different mechanisms [42]. As there is no experimental 3D structure of any anhydro-Sia transporter at present, we show here AlphaFold2 [32] predictions to emphasise the major structural differences among these four groups (the AlphaFold2 prediction of the SatXYZ transporter includes the multitask ATPase, MsmK [44], to complete the functional complex; all AlphaFold2 predictions were visualised with CCP4mg [45]). Anhydro-Sia released by IT-sialidases may be either 2,7-anhydro-Neu5Ac (2,7-AN) or 2,3-dehydro-2-deoxy-Neu5Ac (Neu5Ac2en); Neu5Ac is shown as the β-anomer (ca 90 % at equilibrium). Experimentally confirmed uptake pathways are limited to those using *Rg*SatXYZ and *Ec*NanX (arrows), both for 2,7-AN acquisition. NanOx (known as NanY in *E. coli*) is the cytoplasmic oxidoreductase which performs the obligate step of converting anhydro-Sia into Neu5Ac [7,21], which then can be catabolised by the conserved Nan and Nag enzymes [4]. CM: cytoplasmic membrane.

Sialic acid (Sia) covers a family of over 50 chemically and structurally related nine-carbon sugar acids ubiquitous across vertebrates [1]. Sia residues cap the glycan chain of glycoproteins and glycolipids found on the cell surface of mucosal tissues (such as those lining the respiratory, gastrointestinal, and urogenital tracts), where they mediate key biological interactions based on glycan-protein recognition, regulating numerous processes including immune system functions and neural development [1,2]. N-acetyl-neuraminic acid (Neu5Ac; Fig. 1) is the most abundant Sia in nature and the only form synthesised *de novo* by humans [3]. Bacteria living in mucosal surfaces can access terminal Sia for their own benefit [4,5] by incorporating Sia into own cell surface structures (such as capsules and lipopolysaccharides) [5,6] to evade the immune system by ‘molecular mimicry’ [5] and/or through consumption of Sia for growth to enhance colonisation of established niches [7,8]. The release of Sia from host surfaces by sialidases and its subsequent uptake into the cell by Sia transporters are essential steps in these processes [4]. Most bacterial sialidases belong to glycoside hydrolase family 33 (GH33) of the CAZy database (www.cazy.org [9]), where they liberate monomeric Neu5Ac from a variety of glycans [4,10]. In addition intramolecular *trans* (IT)-sialidases from GH33 have been shown to release anhydro-forms of Neu5Ac (Fig. 1) upon cleavage through an intramolecular *trans*glycosylation reaction, leading to 2,7-anhydro-Neu5Ac (2,7-AN) [11,12] made by, e.g. *Streptococcus pneumoniae* D39 NanB [11,13] and *Ruminococcus gnavus* ATCC 29149 NanH [12], or 2,3-dehydro-2-deoxy-Neu5Ac (Neu5Ac2en) made by, e.g. *S. pneumoniae* TIGR4 NanC [14]. The importance of Neu5Ac and 2,7-AN metabolism in niche colonisation has been established in experimental models [4,7, 15], while Neu5Ac2en metabolism has only been studied *in vitro* using bacterial cultures [16] and/or purified enzymes [14,16].

To acquire Sia, bacteria employ specific transporters located in the cytoplasmic membrane [17,18]. Numerous types of Sia transporters have been identified, reflecting the ecological importance of this trait in Sia-rich niches [17]. Sia transporters belong to four superfamilies of prokaryotic transporters with major differences in fold, subunit composition, and mode of energisation [18]: the MFS (major facilitator superfamily), SSS (sodium-solute symporter), ABC (ATP-binding cassette), and TRAP (tripartite ATP-independent periplasmic transporter) superfamilies. Phylogenetic differences within these groups split Sia transporters into eight independently evolved families (‘ST1-8’) which differ by finer structural-functional properties [17]. A key difference among Sia transporters is their substrate specificity for Neu5Ac and/or 2,7-AN/Neu5Ac2en [17]. Decades of research on Neu5Ac transporters have identified six experimentally confirmed ST families (ST1-6) [17] and provided detailed genetic and mechanistic studies [4,17] alongside some experimental 3D structures [4,19, 20]. In contrast, much less is known on anhydro-Sia transporters (Fig. 1). The ABC transporter SAT2 from *R. gnavus* ATCC 29149 belonging to the ST7 family (renamed SatXYZ in [17]) has been shown to be specific for 2,7-AN using both biochemical and genetic approaches [7]. Three other types of anhydro-Sia transporters have been identified in recent bioinformatics analyses [17,21] based on the conserved genetic link with the oxidoreductase NanOx (NanY in *Escherichia coli*), which is required to convert anhydro-Sia into Neu5Ac for further metabolism into the cells [7,16, 21]. NanX (belonging to the ST1/MFS family), found in *E. coli* K12 and other Gram-negative bacteria, has been confirmed to transport anhydro-Sia, but not Neu5Ac, using *E. coli* BW25113 genetics and growth experiments [16,21]. Instead, NanG transporters (belonging to the ST8/MFS family), found in some *Firmicutes* (*Bacillota*), and orthologues of the widespread SiaX transporters (belonging to the ST5/SSS family), also found in *Bacillota*, remain uncharacterised [17].

Here, we used an improved functional expression system based on a fully Sia-transport-null mutant *E. coli* strain to provide experimental evidence for the substrate preference of the previously uncharacterised NanG and SiaX anhydro-Sia transporters.

## Methods

### Strains and plasmids

Strains and plasmids are listed in Table 1, oligonucleotides are listed in Table S1. Details of strains and plasmids construction are found in Supplementary Methods.

**Table 1.** Strains and plasmids used in this study

NAME	RELEVANT GENOTYPE*	Source
**Strains**		
TRXC2	BW25113(Δ*nanT*::FRT,Δ*nanX*::F3,Δ*nanR*,Δ*nagC*)†,‡	This work
JW5769	BW25113(Δ*nanY*::FRT-Kan^R^-FRT)	[21,46]
**Plasmids**		
pWKS30	Low-copy cloning vector with *lac* promoter and pSC101 *ori*	[47]
pES1G	pWKS30 +*nanT* from *Escherichia coli* BW25113	[48]
pES156	pWKS30 +*nanXY* from *Escherichia coli* BW25113	[21]
pES21	pWKS30 +*nanX* from *Escherichia coli* BW25113(ESX1 +ESX2)	This work
pDRT1	pWKS30 +*nanG* (*B5P37_01885*) from *Staphylococcus lutrae* DSM 10244(ESN601 +ESN602)	This work
pDRT2	pWKS30 +*siaX* (*B5P37_10005*) from *Staphylococcus lutrae* DSM 10244(ESN603 +ESN604)	This work
pDRT4	pWKS30 +*SlnanG*-TEV-His6	This work
pDRT5	pWKS30 +*SlnanG*[D40A]-TEV-His6	This work
pDRT7	pWKS30 +*nanX* (*STM1132*) from *Salmonella typhimurium* LT2(ESN617 +ESN618)	This work
pES41	pWKS30 +*siaT* (*STM1128*) from *Salmonella typhimurium* LT2	[22]
pSEV21	pWKS30 +*siaT* (*LV622_01385*) from *Staphylococcus aureus* RN6390(ESN358 +ESN359)	This work
pSEV33	pWKS30 +*siaX* (*FF104_02255*) from *Clostridium butyricum* ATCC 19398(ESN437 +ESN438)	This work
pKD46	λRed recombineering functions under *araBAD* promoter control	[49]
pCP20	FLP recombinase expression	[49]
pES101	pUC57kan-F3-*aadA* (Spec^R^)-F3‡	This work
pKD13	FRT-*nptII* (Kan^R^)-FRT template plasmid	[49]
pES134	Same as pKD13, but with F3-*aadA*-F3‡	This workAddgene 127 551

*Names in parentheses refer to the primers used to amplify those Sia transporter genes that were cloned by restriction-ligation (see Supplementary Methods for details and Table S1 for primer sequences). In all cases genomic DNA (gDNA) was used as template. All gDNA samples were made in-house except for that of *S. lutrae* DSM 10244, which was purchased from DSMZ.

†The *nanR* and *nagC* genes code for the transcriptional repressors of the *nan* and *nag* regulons, respectively [25,28], hence their deletion results in the constitutive expression of the *nan* and *nag* genes required for Neu5Ac catabolism [28]. See Supplementary Methods for the details of TRXC2 construction.

‡F3 are orthogonal variants of the FRT sites (see Supplementary Methods).

### Growth experiments

**Fig. 2.**
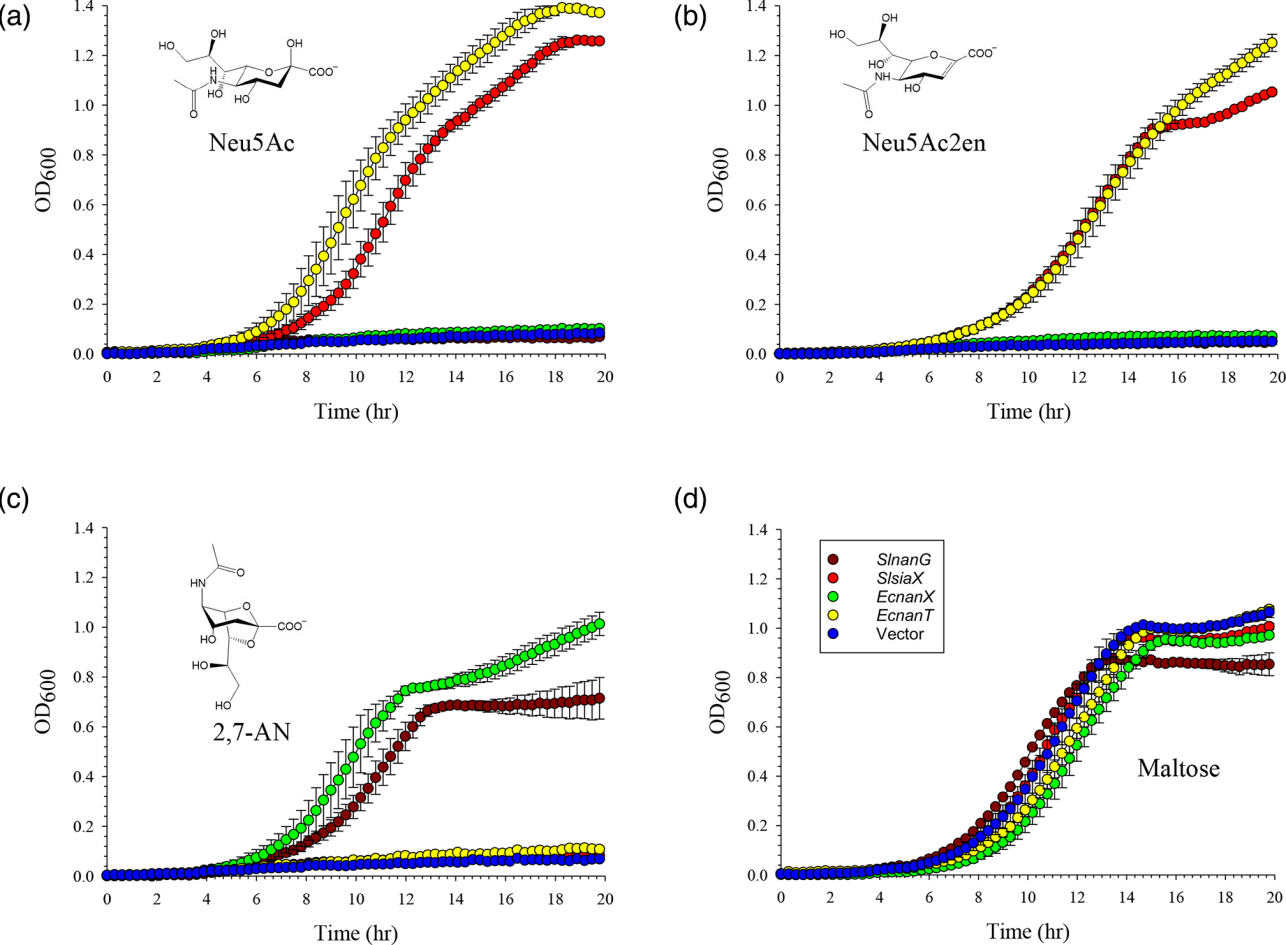
Complementation of the Sia-transport-null strain of *E. coli*, TRXC2, with anhydro-Sia transporter genes. Plasmids carrying different Sia transporter genes under *lac* promoter control were introduced into TRXC2 and the resulting cultures tested for growth in minimal M9 medium with different Sias (structures in the insets) as sole carbon source. Growth experiments were performed in the presence of 1 mg ml^−1^ (≈ 3.5 mM) of either Neu5Ac (**a**), Neu5Ac2en (**b**), 2,7-AN (**c**), or maltose (**d**), the latter used as a control for Sia-independent growth. Red: *SlsiaX*; brown: *SlnanG*; green: *EcnanX* (positive control for 2,7-AN growth); yellow: *EcnanT* (positive control for Neu5Ac growth); blue: empty vector, pWKS30. Data are the average from triplicate sets±SD.

For growth experiments using minimal medium, overnight starter cultures of transformed TRXC2 strains were prepared as described previously [22] in M9 medium [23] supplemented with 125 μg ml^−1^ ampicillin (‘M9Amp’) and 0.4 % v/v glycerol at 37 °C. These were diluted in M9 salts to 10× the required initial OD_600_ (see below) and eventually diluted into experimental M9Amp medium supplemented with 0.1 mM IPTG and carbon source at 0.2–1 mg ml^−1^ (ca 0.7–3.5 mM – see main text for details). Carbon sources were: maltose (Alfa Aesar), replacing glucose [22] for control growth curves, Neu5Ac (Fluorochem), 2,7-AN (made in-house as in [24]), and Neu5Ac2en (Merck). For our initial experiment (Fig. 2) the initial OD_600_ was set at 0.001, but for all later experiments this was increased to 0.01 to shorten the apparent lag phase. Growth was performed in a TECAN Infinite M Nano^+^ plate reader at 37 °C with shaking every 5 min and sampling every 20 min for 20 h. JW5769 transformants were processed in the same manner except that the starter cultures were grown with Neu5Ac 2 mg ml^−1^ instead of glycerol to pre-induce the *nan* regulon [25].

## Results

### Functional expression in *E. coli* of NanG, SiaX, and NanX anhydro-Sia transporters

*E. coli* K12 strains possess two Sia transporter genes, *nanT*, coding for a well-known Neu5Ac transporter [22,26, 27], and *nanX*, coding for an anhydro-Sia transporter [16,21]. Here, we generated an *E. coli* strain (TRXC2; Table 1) which lacks both native transporter genes by introducing a *nanX* deletion into SEVY3 (BW25113Δ*nanT*,Δ*nanR*,Δ*nagC*; [19]). Due to the deletion of the *nanR* and *nagC* repressor genes [19], which control all other Sia-utilisation genes converting Sia into fructose-6-P for glycolysis [25,28], the Sia utilisation pathway in this strain is expressed constitutively; this means that any specific growth defects will reflect the transporter specificity and/or affinity for the substrate [19].

To characterise the anhydro-Sia transporters NanG and SiaX, we selected the examples encoded by the genes *B5P37_01885* (*SlnanG*) and *B5P37_10005* (*SlsiaX*) of *Staphylococcus lutrae* DSM 10244 [29], which were previously identified based on their genetic association with NanOx orthologues showing ≥60 % identity to the experimentally characterised oxidoreductases from *R. gnavus*, *Rg*NanOx, and *E. coli*, *Ec*NanY [7,17, 21]. We tested the substrate specificity of these transporters as well as that of *Ec*NanX and *Ec*NanT as controls by complementation of TRXC2 isogenic strains. When TRXC2 was complemented with *EcnanX*, the bacteria could grow on 2,7-AN (Fig. 2c), while complementation with *EcnanT* enabled growth on both Neu5Ac and Neu5Ac2en (Fig. 2a, b), in agreement with the results using individual *nanT* and *nanX* deletions [21,22]. Complementation with *SlnanG* only supported growth on 2,7-AN, with growth rates and final density comparable to those conferred by *EcnanX* (Fig. 2c). This was also observed using the *nanX* gene (*STM1132*) from *Salmonella typhimurium* LT2 [17,21], which codes for a transporter 57 % identical to *Ec*NanX [17]. *STM1132*-expressing TRXC2 cultures grew only on 2,7-AN (Fig. S1, available in the online version of this article). Finally, *SlsiaX* complementation enabled growth on both Neu5Ac and Neu5Ac2en, but not of 2,7-AN (Fig. 2a, b).

These results provided experimental evidence for the Sia substrate preference of NanG and SiaX transporters, with NanG, like both NanX transporters, being specific for 2,7-AN only, and with SiaX instead being specific for Neu5Ac and Neu5Ac2en, but not for 2,7-AN, as summarised in Table 2. This is the first experimental characterisation of novel MFS and SSS transporters involved in anhydro-Sia acquisition. In addition, we confirmed using an isogenic and previously characterised *nanY* mutant of *E. coli* [21], that growth on 2,7-AN or Neu5Ac2en was dependent on the oxidoreductase *Ec*NanY (Fig. S2), confirming the essential role of this enzyme for anhydro-Sia metabolism.

**Table 2.** Substrate specificity of the anhydro-Sia transporters investigated in this study

	2,7-AN	Neu5Ac2en	Neu5Ac
***Sl*NanG**	+	−	−
***Sl*SiaX**	−	+	+
***Ec*NanX**	+	−	−
**STM1132 (*St*NanX**)	+	−	−

### A potential 2,7-AN-binding site of *Sl*NanG

We next investigated the structural basis for the substrate preference for 2,7-AN by *Sl*NanG. To identify potential functional residues, we performed a DALI search [30] of the PDB database [31] using an AlphaFold2 [32] model of *Sl*NanG (Fig. 1) as query. The two best hits (PDB 7L17 and 7L16, both with 20 % identity to *Sl*NanG and respectively with 2.9–3.0 Å rmsd across 439/440 pairs) were with the crystal structures of sugar-bound *S. typhimurium* MelB, a well-characterised transporter of the GPH (Glycoside-Pentoside-Hexuronide:cation) family [33] (Fig. S3A). The overlay between *Sl*NanG’s AlphaFold2 prediction and entry 7L17 (*St*MelB bound with 4-nitrophenyl alpha-d-galactopyranoside, an analogue of the native substrate melibiose) identified *Sl*NanG D40 (a conserved residue within the NanG/ST8 family; Fig. S3B) as the equivalent of MelB D19 (Fig. S3A), which is a critical substrate-binding site residue forming key H-bonds with melibiose [33,34].

**Fig. 3.**
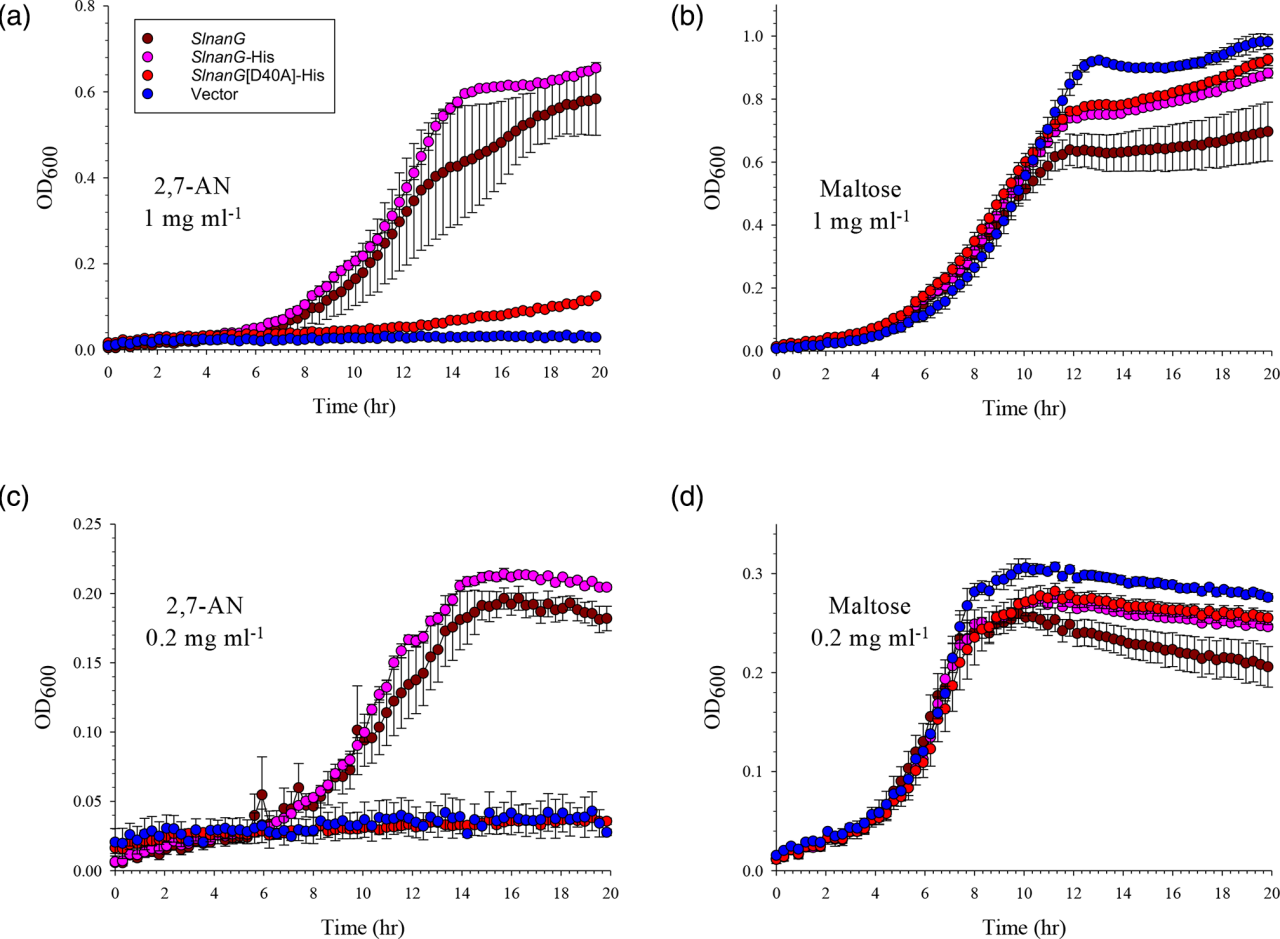
D40 of *Sl*NanG is required for high-affinity uptake of 2,7-AN. Plasmids carrying different alleles of *SlnanG* were introduced into TRXC2 and tested for their ability to complement the growth of this strain on ‘high’ (1 mg ml^−1^ ≈ 3.5 mM; **a**) and ‘low’ (0.2 mg ml^−1^ ≈ 0.7 mM; **c**) concentrations of 2,7-AN. (b) and (d): same as in (a) and (c), respectively, but with maltose as control carbon source. Brown: WT *SlnanG*; pink: *SlnanG*-His, coding for a C-terminally His_6_-tagged variant; red: *SlnanG*[D40A]-His; blue: empty vector, pWKS30. Data are the average from triplicate sets±SD.

To investigate the contribution of D40 to NanG function, we generated a *SlnanG* D40A mutant and tested it for complementation of TRXC2 on high (ca. 3.5 mM) and low (ca. 0.7 mM) 2,7-AN concentrations to exacerbate differences. The mutant showed no growth on low 2,7-AN, and poor growth on high 2,7-AN concentration (Fig. 3). The mutation had no effect on protein levels in the *E. coli* cytoplasmic membrane (Fig. S3C). These data are consistent with D40 being a key functional residue of NanG transporters for substrate-binding to 2,7-AN.

### Evidence for distinct substrate specificities in ST5 Sia transporters

SiaX and SiaT transporters form two separate clades of otherwise related Sia transporters (ST5) which have been extensively studied for their capacity to transport Neu5Ac [17,22, 35, 36] and share Neu5Ac-binding site [17,36]. Our data showed that *Sl*SiaX, which forms a genetic pair with a NanOx/NanY protein (encoded by the *B5P37_10015* gene [17]), can transport both Neu5Ac2en and Neu5Ac, and that growth on Neu5Ac2en was enabled by *Ec*NanY. However, not all SiaX orthologues are genetically linked to NanOx [17]. Here, we used the approach described above to gain more insights into Neu5Ac2en transport within the ST5 family by targeting ST5 genes unlinked to NanOx, namely, *siaX* (*FF104_02255*) from *Clostridium butyricum* ATCC 19398, and the *siaT* genes from *S. typhimurium* LT2 (*STM1128*) and *Staphylococcus aureus* RN6390 (*LV622_01385*) [37], both coding for characterised Neu5Ac transporters [22,35].

**Fig. 4.**
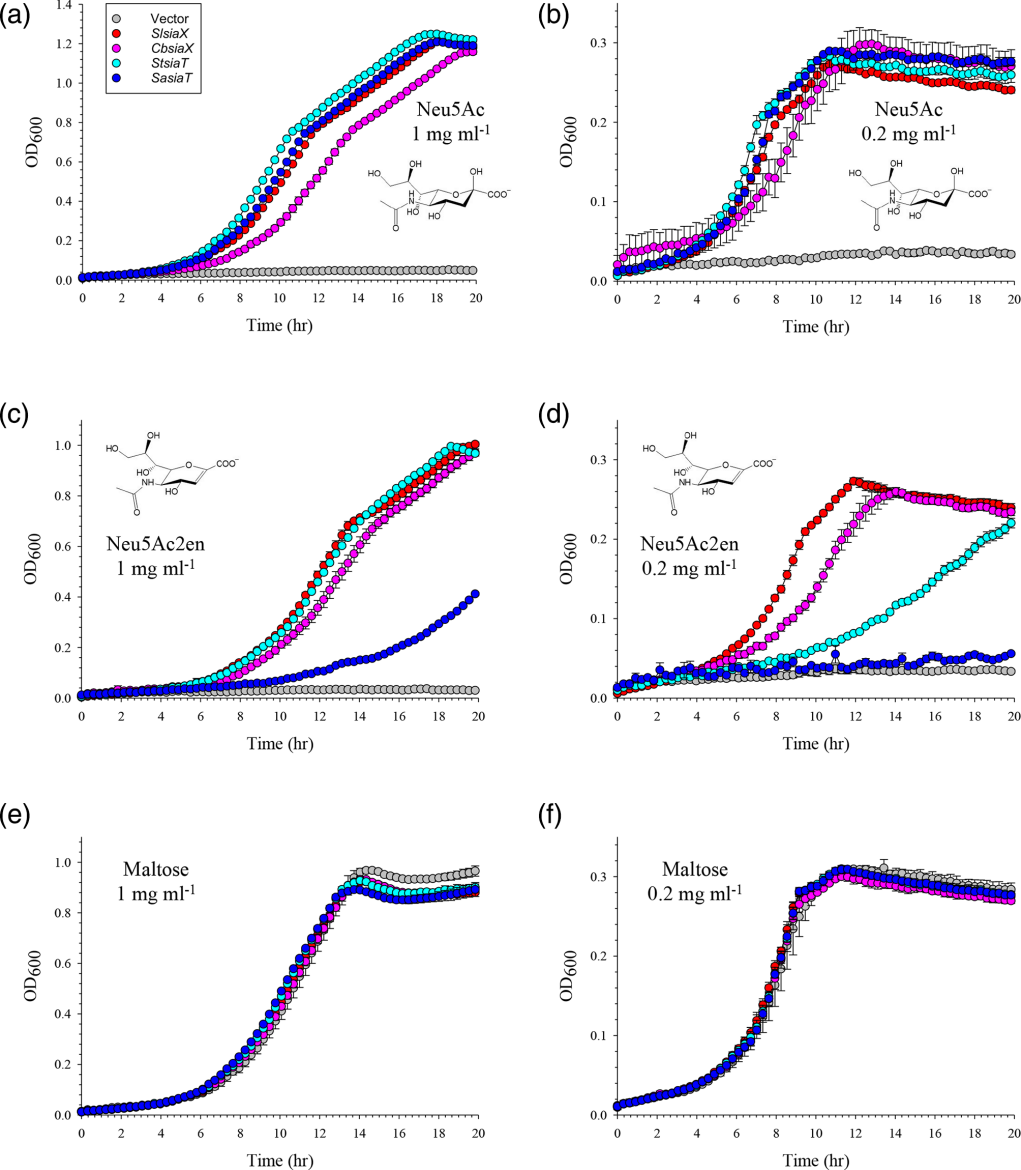
SiaX and SiaT transporters of the ST5 family compared for their ability to take up Sias. Plasmids carrying different ST5 transporter genes were introduced into TRXC2 and tested for their ability to complement the growth of this strain on different substrates at ‘high’ (1 mg ml^−1^ ≈ 3–3.5 mM; a, c, and e) and ‘low’ (0.2 mg ml^−1^ ≈ 0.6–0.7 mM; b, d, and f) concentrations. (a) and (b), Neu5Ac; (c) and (d), Neu5Ac2en; (e) and (f), maltose (control). Red: *SlsiaX*; pink: *C. butyricum* (*Cb*) *siaX*; cyan: *S. tytphimurium* (*St*) *siaT*; blue: *S. aureus* (*Sa*) *siaT*; grey: empty vector, pWKS30. Data are the average from triplicate sets±SD.

All isogenic strains grew comparably well on Neu5Ac and maltose irrespective of substrate concentration and transporter expressed (Fig. 4a, b, e, f), but complementation with the *siaX* genes outperformed that with their *siaT* orthologues in enabling growth on Neu5Ac2en, with growth profiles for Neu5Ac2en resembling those on Neu5Ac even at low concentration of substrate (Fig. 4c, d). These results are consistent with the capacity of SiaX transporters, but not of SiaT transporters, to scavenge both Neu5Ac2en and Neu5Ac, and support the role of NanOx in this process.

## Discussion

Bioinformatic analyses identified four types of Sia transporters involved in anhydro-Sia uptake based on their genetic association with the anhydro-Sia oxidoreductase, NanOx/NanY [17,21] (Fig. 1). Of these, *Rg*SatXYZ/SAT2 (ST7/ABC) and *Ec*NanX (ST1/MFS) were previously shown to transport 2,7-AN [7,16, 21]. Here we provided the first experimental evidence for the substrate preference of NanG (ST8/MFS) and SiaX (ST5/SSS) transporters by expressing the *SlnanG* or *SlsiaX* genes in a Sia-transport-null mutant of *E. coli* without interference from the endogenous Sia transporter genes, *EcnanT* and *EcnanX*. We showed that NanG could only transport 2,7-AN, while SiaX was a promiscuous Neu5Ac/Neu5Ac2en transporter.

Previous studies using genetic complementation of single-transporter mutants of *E. coli* showed that *Ec*NanX could transport 2,7-AN only [21], or both 2,7-AN and Neu5Ac2en [16]. Here we showed that, when expressed as the sole Sia transporter in the Sia-transport-null *E. coli* mutant, *Ec*NanX was specific for 2,7-AN, while *Ec*NanT could transport both Neu5Ac and Neu5Ac2en, which is in line with the structural homology of Neu5Ac2en to Neu5Ac but not to 2,7-AN (Fig. 1). In addition, we showed here that a different NanX transporter, the *STM1132* gene product [17,21], also exclusively transported 2,7-AN, further supporting this substrate specificity within the NanX group. Future biochemical and structural studies of NanX orthologues [17] are warranted to ascertain the 2,7-AN specificity of NanX transporters.

ST5 transporters including some well-characterised SiaT proteins [22,35, 36, 38] and the SiaX transporter from *C. difficile* [39,40] transport Neu5Ac in their native organisms. The capacity of SiaX proteins from this family (ST5) to take up Neu5Ac2en efficiently and the ability of NanOx oxidoreductases to convert Neu5Ac2en into Neu5Ac [16] mean that this substrate may support bacterial growth even in limiting concentrations. The sporadic nature of the SiaX-NanOx genetic association [17] suggests however that this might be a partnership of ‘convenience’ which exploits naturally promiscuous transporters to tap into the local pool of Neu5Ac2en. Indeed, we found that both NanOx-linked *Sl*SiaX and ‘free’ *Cb*SiaX could transport Neu5Ac2en efficiently. Our results from comparing SiaX transporters with the well-known SiaT proteins from *S. typhimurium* LT2 and *S. aureus* RN6390, showed that the latter are poor Neu5Ac2en transporters, and might explain our previous finding that NanOx does not form a partnership with proteins from this group of the ST5 family [17]. Mutagenesis and structural studies are wanted to tease out the details of substrate recognition by different ST5 transporters and to understand the molecular bases of the in-built promiscuity of SiaX transporters.

Overall, our study completed the characterisation of the four types of anhydro-Sia transporters in bacteria. Future studies are needed to investigate the function of the novel transporters, NanG and SiaX, in native organisms to understand the significance of these Sia pathways in host-microbe and microbe-microbe interactions in health and disease.

## supplementary material

10.1099/mic.0.001448 Uncited Supplementary Material 1.
