# Corrigendum: Characterisation of anhydro-sialic acid transporters from mucosa-associated bacteria

**DOI:** 10.1099/mic.0.001476

**Published:** 2024-08-02

**Authors:** Yunhan Wu, Andrew Bell, Gavin H. Thomas, David N. Bolam, Frank Sargent, Nathalie Juge, Tracy Palmer, Emmanuele Severi

**Affiliations:** 1 Microbes in Health and Disease, Biosciences Institute, Newcastle University, Framlington Place, Newcastle upon Tyne NE2 4HH, UK; 2 Quadram Institute Bioscience, Gut Microbes and Health Institute Strategic Programme, Rosalind Franklin Road, Norwich Research Park, Norwich NR4 7UQ, UK; 3 Department of Biology and York Biomedical Research Institute (YBRI), Wentworth Way, University of York, York YO10 5DD, UK

In Figure S1, panel B, which should have illustrated the growth of complemented strains on Neu5Ac2en and instead was an accidental duplication of panel A for growth on Neu5Ac, has now been replaced to report the correct graph for growth on Neu5Ac2en. Results and conclusions are unaffected.

**Figure S1. NanX from *Salmonella typhimurium* LT2 is a 2,7-AN-specific transporter.** Plasmids carrying different *nanX* genes (Table 1) were introduced into TRXC2 and tested for their ability to complement the growth of this strain on different Sias (all used at 1 mg ml^−1^ ≈ 3.5 mM) as sole carbon source. A: Neu5Ac; B: Neu5Ac2en; C: 2,7-AN; D: maltose. Light green: *EcnanX*; dark green: *STM1132* (*nanX* from *S. typhimurium* LT2); yellow: *EcnanT* (positive control for growth on Neu5Ac, negative control for growth on 2,7-AN). Data are the average from triplicate sets±SD.

The Authors apologise for any inconvenience caused.

## supplementary material

10.1099/mic.0.001476 Uncited Fig. S1.

