# Supplementary material for: Characterisation of anhydro-sialic acid transporters from mucosa-associated bacteria
Source: Microbiology (Reading). 2024 Mar 15;170(3):001448. doi: 10.1099/mic.0.001448 (PMC10955332; doi:10.1099/mic.0.001448)
Supplement: Uncited Supplementary Material 1. [file mic-170-01448-s001.pdf]

Characterisation of anhydro-sialic acid transporters from mucosa-associated bacteria

Yunhan Wu[1], Andrew Bell[2], Gavin H. Thomas[3], David N. Bolam[1], Frank Sargent[1], Nathalie Juge[2], Tracy Palmer[1], and Emmanuele Severi[1]*.

1: Microbes in Health and Disease, Biosciences Institute, Newcastle University, Framlington Place, Newcastle upon Tyne NE2 4HH, United Kingdom.
2: Quadram Institute Bioscience, Gut Microbes and Health Institute Strategic Programme, Rosalind Franklin Road, Norwich Research Park, Norwich NR4 7UQ, United Kingdom.
3: Department of Biology and York Biomedical Research Institute (YBRI), Wentworth Way, University of York, York YO10 5DD.
*: correspondence, emmanuele.severi@newcastle.ac.uk

SUPPLEMENTARY INFORMATION

Supplementary Methods
Supplementary Table S1
Supplementary Figures S1-3
Supplementary References

SUPPLEMENTARY METHODS

Strains and plasmids construction

Verify (PCRBIO) and Herculase II Fusion (Agilent) were used for high-fidelity PCR, while GoTaq G2 (Promega) for colony PCR. PCRs were treated with DpnI before cloning. Gibson assemblies were performed with either the Quick-Fusion (Biotool) or ClonExpress II (Vazyme) cloning kits. XL1-blue (Agilent) and EC100D*pir*+ (Lucigen/Epicentre) were used as cloning strains.

E. coli strain TRXC2 (BW25113Δ*nanT*::FRT,Δ*nanX*::F3,Δ*nanR*,Δ*nagC*,) is a derivative of SEVY3 [1] with the additional in-frame deletion of *nanX*, which was introduced by λRed recombineering [2] followed by marker removal and plasmid curing [3]. The marker cassette, containing the *aadA* (Spectinomycin^R) gene flanked by F3 sites [4], was amplified from pES134 (construction details below) with KEIO oligos ESN361+ESN353 (yjhB-fwd and -rev from [3]). After genetic modification, all mutant loci of TRXC2 were confirmed by PCR (Table S1) and the new mutation also by sequencing. Strain JW5769 (BW25113Δ*nanY*::FRT-Kan^R-FRT) from the KEIO collection was used without further modification, as done previously [5].

The pKD13-type template plasmid, pES134, was made by Gibson assembly of PCR products ES184+ES185 for the marker cassette, amplified from the synthetic construct pES101 (GenScript), and ES186+ES187 for the backbone, amplified from pKD13. pES134 reproduces the design of pKD13 [2] and may be used in the same way to introduce unmarked in-frame deletions in the *E. coli* chromosome [3] without illegitimate recombination with pre-engineered FRT scars because of FRT-F3 orthogonality [4]. pES134 is available from Addgene (127551).

Constructs pDRT1, pDRT2, pDRT7, pES21, pSEV21, and pSEV33, all isogenic derivatives of pWKS30 [6] carrying different sialic acid transporter genes under *lac* promoter control, reproduce the design of pES1G, pES41, and pES156, described previously [5, 7, 8], and were made in an equivalent manner by restriction-ligation of PCR products amplified from genomic DNAs (Tables 1 and S1). pDRT4 was made by Gibson assembly of PCR products ESN257+ESN607 for *nanG* (entire coding sequence minus stop

codon), amplified from pDRT1, and ESN255+ESN606 for the backbone, amplified from pSEV7 (pSEV7 is a derivative of pWKS30 coding for C-terminal TEV site and His_6 tag adding sequence - ENLYFQGLEHHHHHH to the last residue of the encoded transporter; Eunice Lee and Emmanuele Severi, unpublished). pDRT5 was made analogously, with the *nanG*-[D40A] insert amplified as two overlapping PCR products (ESN257+ESN612 and ESN613+ESN607). All constructs were verified by sequencing using oligos ESN102 and ESN103 [9].

Cell fractionation and western blotting. Transformed TRXC2 strains were precultured as described for the growth experiments, refreshed to an initial OD_{600} of 0.1 in M9Amp supplemented with 0.1 mM IPTG and 2 mg ml^{-1} glucose, and grown to ca 1 OD_{600}. Cells were then harvested and subjected to cellular fractionation as described in [9] to separate the membrane fraction, samples of which were then probed with anti-His antibody (Invitrogen) as per the same reference.

Structural comparison between *Sl*NanG and *St*MelB

An the AlphaFold2 model of *Sl*NanG was generated *via* the Colaboratory online tool [10] and the "rank_001" model was used to search the entire PDB database [11] using the DALI server [12]; both online tools were used with default parameters. The top two hits were the two experimental structures of sugar-bound *Salmonella typhimurium* LT2 MelB [13], namely entries 7L16 (MelB bound to dodecyl 6-O-alpha-D-galactopyranosyl-beta-D-glucopyranoside; 20% identity, 3 Å rmsd across 440 pairs) and 7L17 (MelB bound to 4-nitrophenyl alpha-D-galactopyranoside; 20% identity, 2.9 rmsd across 439 pairs). To identify a potential substrate-binding site within *Sl*NanG, we superimposed the AlphaFold2 model of *Sl*NanG with entry 7L17 using ChimeraX [14], which was also used to visualise the resulting overlay.

Table S1

NAME	SEQUENCE 5´→3´[a]	USE[b]
ESN102	TGTAAAACGACGGCCAGT	SEQ
ESN103	CAGGAAACAGCTATGAC	SEQ
E193	CCTCGCTTTGTAACGGAGTAGAG	SEQ
E194	GACGGATGGCCTTTTTGCGTGGC	SEQ
ESN331	CTGGTAATAAACTGCGCCATG	DIAG
ESN332	CATCTGATAGAGATCGCCAG	DIAG
ESN333	GAAAACGCCGACGTCATTAC	DIAG
ESN334	GAATATCGGTACGCAGGTTATC	DIAG
ESN351	CGCAAACCGTTTGGACCGGTAG	DIAG
ESN352	CACCTTCAATGCGAATGGCAAC	DIAG
ESN354	GTTGGTGACCCAAAACTGTAG	DIAG
ESN355	GCTACGATCACGCAATCGAC	DIAG
ESN353	AACGCCATAATTAATCATAAAGCCTCCAATCATTTAGCCACGGATAGTTTTGTAGGCTGGAGCTGCTTCG	RED
ESN361	CTAATATTTACTTTAAGGGCTATATTAGAATAACACAGGAAACAAATATGATTCCGGGGATCCGTCGACC	RED
E184	ATTCCGGGGATCCGTCGACC	GIBS
E185	TGTAGGCTGGAGCTGCTTCG	GIBS
E186	GGTCGACGGATCCCCGGAAT	GIBS
E187	CGAAGCAGCTCCAGCCTACA	GIBS
ESN255	CATATGTATATCTCCTTCTTTTACGG	GIBS
ESN257	CCGTAAAAGAAGGAGATATACATATG	GIBS
ESN606	GAGAATTTATATTTTCAAGGCCTCG	GIBS
ESN607	CGAGGCCTTGAAAATATAAATTCTCTGCATTAACCTGTAACCTTTCAC	GIBS
ESN612	CGAGAATCGCAGCATTACCAATG	GIBS
ESN613	CATTGGTAATGCTGCGATTCTCG	GIBS
ESX1	GCGGTACCGTAAAAGAAGGAGATATACAT**ATG**GCAACAGCATGGTATAAAC	RLC
ESX2	GCGGATCC*TCA*TTTAGCCACGGATAGTTTA	RLC
ESN358	GATGCAACTGGTCTCGGTACCGTAAAAGAAGGAGATATACAT**ATG**AAAGAAGTTGGATTTGGC	RLC
ESN359	CTGACGCATGGTCTCGGATCC*CTA*CTTTTTCGTAGCCGTTTTTG	RLC
ESN437	GATGCAACTGGTCTCGGTACCGTAAAAGAAGGAGATATACAT**ATG**CAAGGTTTTACAAAAATAGATTTAG	RLC
ESN438	CTGACGCATGGTCTCGGATCC*TTA*TTTGTTATATATAGTTGCATCCATATTAG	RLC
ESN601	CAGTGAGTCGTCTCGGTACCGTAAAAGAAGGAGATATACAT**ATG**AAACAAAAAGAAGGTGCAGTTTC	RLC
ESN602	CCTAGTTCCGTCTCGGATCC*TCA*TGCATTAACCTGTAACCTTTC	RLC
ESN603	CAGTGAGTCGTCTCGGTACCGTAAAAGAAGGAGATATACAT**ATG**AAAACAACGGGTTTTACTTTAATC	RLC
ESN604	CCTAGTTCCGTCTCGGATCC*TTA*ATGTCGTTTTTCCCAAGAACC	RLC
ESN617	CAGTGAGTCGTCTCGGTACCGTAAAAGAAGGAGATATACAT**ATG**ATAGCAAAATTCTTCCCGTG	RLC
ESN618	CCTAGTTCCGTCTCGGATCCT*GTA*CCTACAACACCGTATTTCG	RLC

a = restriction sites used for cloning (Acc65I, BamHI, and BsaI and EspI sites releasing compatible ends with the former two) are underlined. Start and stop codons of Sia transporter genes are in bold and in italics, respectively. The mutagenic codon for *SlnanG*[D40A] is highlighted in grey. Please note that oligo ESN618, used to amplify *STM1132* (*nanX*) from *S. typhimurium* LT2, anneals downstream of that gene into the 5´ end of *STM1133* (*nanY*/*nanOx*) adding an in-frame stop codon.

b = SEQ: used for sequencing (ESN102/ESN103 for pWKS30 inserts, E193/E194 for pKD13 inserts); DIAG: diagnostic PCR primers for the gene deletion loci in strain TRXC2 (ESN331/ESN332, Δ*nanR*; ESN333/ESN334, Δ*nagC*; ESN351/ESN352, Δ*nanT*::FRT; ESN352/ESN354, Δ*nanX*::F3); GIBS: used for Gibson assembly; RLC: used for restriction-ligation cloning (see Table 1 for construct details).

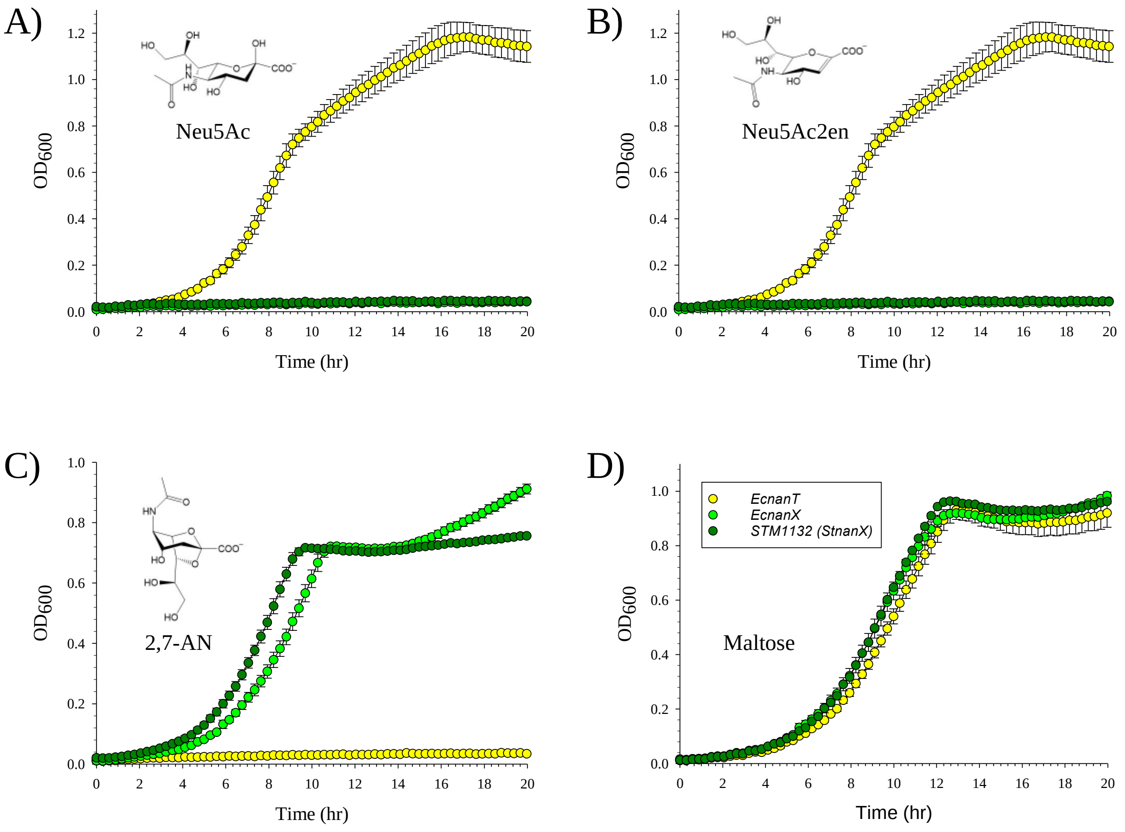

Figure S1. NanX from *Salmonella typhimurium* LT2 is a 2,7-AN-specific transporter. Plasmids carrying different *nanX* genes (Table 1) were introduced into TRXC2 and tested for their ability to complement the growth of this strain on different Sias (all used at 1 mg ml^{-1} ≈ 3.5 mM) as sole carbon source. A: Neu5Ac; B: Neu5Ac2en; C: 2,7-AN; D: maltose. Light green: *EcnanX*; dark green: *STM1132* (*nanX* from *S. typhimurium* LT2); yellow: *EcnanT* (positive control for growth on Neu5Ac, negative control for growth on 2,7-AN). Data are the average from triplicate sets ± SD. *STM1132* codes for a transporter 57% identical to *Ec*NanX [15].

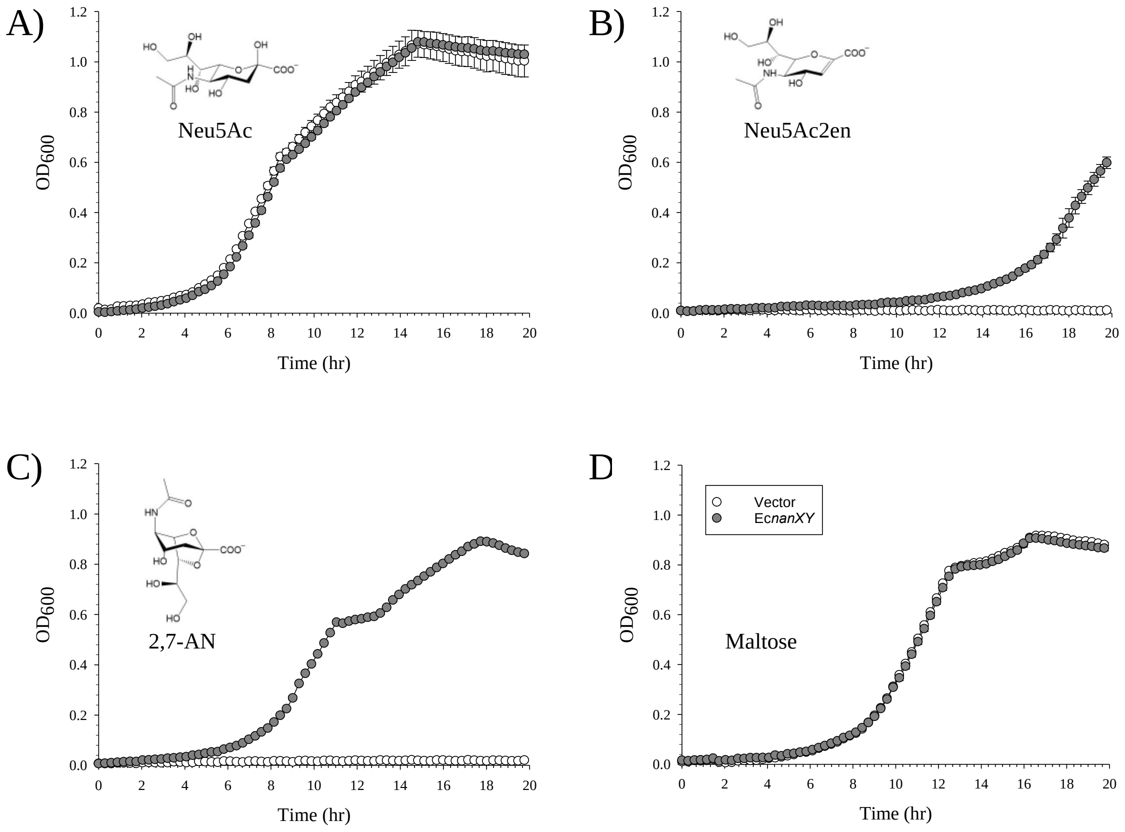

Figure S2. Growth of *E. coli* on anhydro-Sia is dependent on the oxidoreductase NanY. Strain JW5769 (BW25113 Δ*nanY*::FRT-Kan^{R}-FRT) was transformed with a plasmid carrying the *EcnanXY* operon (Table 1) and tested for growth on different Sias (all used at 1 mg ml^{-1} ≈ 3.5 mM) as sole carbon source. A: Neu5Ac; B: Neu5Ac2en; C: 2,7-AN; D: maltose. Grey: JW5769 with *EcnanXY*; white: JW5769 carrying empty vector, pWKS30. Data are the average from triplicate sets ± SD.

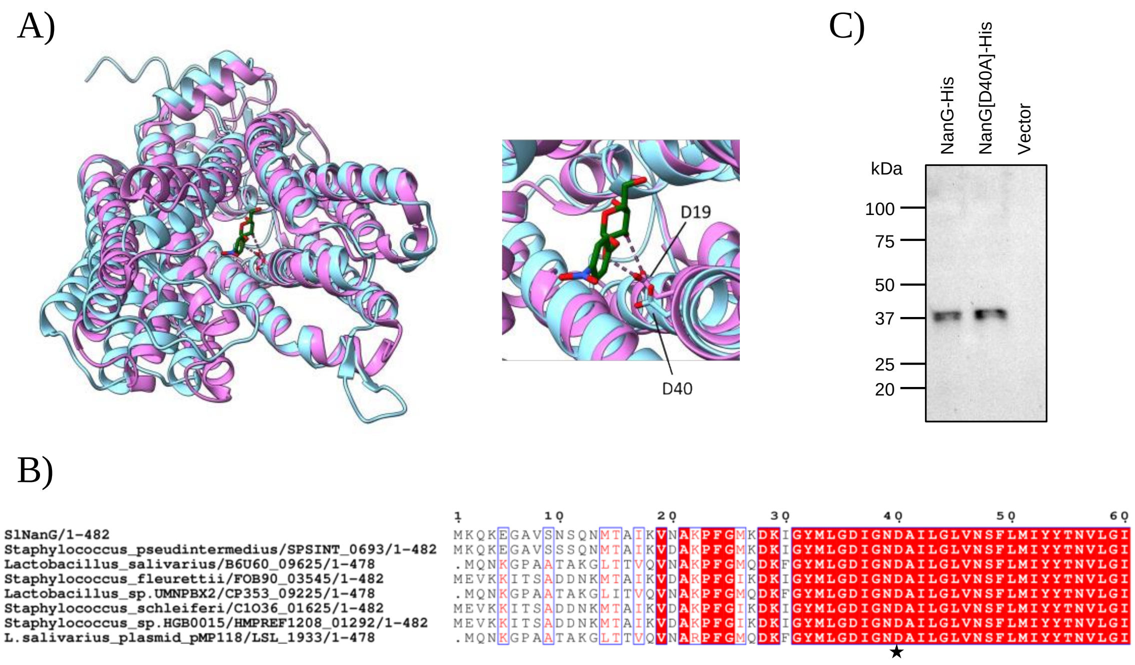

Figure S3. Identification of *Sl*NanG D40 as a functional residue for 2,7-AN uptake. A. Overlay between the AlphaFold2-predicted structure of *Sl*NanG (cyan) and the experimental structure (PDB 7L17) of the melibiose transporter MelB (pink) from *Salmonella typhimurium* (note that AlphaFold2 predicts *Sl*NanG to have 12 transmembrane helices, and not 11 as previously reported [16]). View from the extracytoplasmic side of the transporters into their substrate-binding cavities. Inset: zoom-in on MelB D19 showing the H-bonds with the co-crystallised ligand (the melibiose analogue, 4-nitrophenyl α-D-galactopyranoside) and pinpointing the equivalence of this residue with *Sl*NanG D40; B: Espript [17] alignment (first ~ 60 residues only) of all NanG transporters of the ST8 family [16], with the asterisk highlighting D40 of *Sl*NanG; C: anti-His-tag immunoblot of membrane fractions from *E. coli* TRXC2 expressing either *Sl*NanG-His_6 (from pDRT4), the D40A mutant of the same variant (from pDRT5), or no heterologous transporter at all (from pWKS30, "vector"). Notably, *Sl*NanG migrates as a considerably smaller species (37 rather than the predicted 55 kDa), as seen for other membrane proteins [18].

SUPPLEMENTARY REFERENCES

1. **Peter MF, Ruland JA, Depping P, Schneberger N, Severi E, *et al.*** Structural and mechanistic analysis of a tripartite ATP-independent periplasmic TRAP transporter. *Nat Commun* 2022;13:4471.
2. **Datsenko KA, Wanner BL**. One-step inactivation of chromosomal genes in Escherichia coli K-12 using PCR products. *Proceedings of the National Academy of Sciences* 2000;97:6640–6645.
3. **Baba T, Ara T, Hasegawa M, Takai Y, Okumura Y, *et al.*** Construction of Escherichia coli K-12 in-frame, single-gene knockout mutants: the Keio collection. *Mol Syst Biol* 2006;2:2006.0008.
4. **Branda CS, Dymecki SM, Cre- S**. Talking about a Revolution: Review The Impact of Site-SpecificRecombinases on Genetic Analyses in Mice. 2004;6:1–22.
5. **Bell A, Severi E, Lee M, Monaco S, Latousakis D, *et al.*** Uncovering a novel molecular mechanism for scavenging sialic acids in bacteria. *Journal of Biological Chemistry* 2020;295:13724–13736.
6. **Rong Fu Wang, Kushner SR**. Construction of versatile low-copy-number vectors for cloning, sequencing and gene expression in Escherichia coli. *Gene* 1991;100:195–199.
7. **Mulligan C, Geertsma ER, Severi E, Kelly DJ, Poolman B, *et al.*** The substrate-binding protein imposes directionality on an electrochemical sodium gradient-driven TRAP transporter. *Proceedings of the National Academy of Sciences* 2009;106:1778–1783.
8. **Severi E, Hosie AHF, Hawkhead JA, Thomas GH**. Characterization of a novel sialic acid transporter of the sodium solute symporter (SSS) family and in vivo comparison with known bacterial sialic acid transporters. *FEMS Microbiol Lett* 2010;304:47–54.
9. **Severi E, Bunoro Batista M, Lannoy A, Stansfeld PJ, Palmer T**. Characterization of a TatA/TatB binding site on the TatC component of the Escherichia coli twin arginine translocase. *Microbiology (Reading)* 2023;169:1–18.
10. **Bisong E**. *Building Machine Learning and Deep Learning Models on Google Cloud Platform*. Berkeley, CA: Apress; 2019. Epub ahead of print 2019. DOI: 10.1007/978-1-4842-4470-8.
11. **Burley SK, Bhikadiya C, Bi C, Bittrich S, Chao H, *et al.*** RCSB Protein Data Bank (RCSB.org): delivery of experimentally-determined PDB structures alongside one million computed structure models of proteins from artificial intelligence/machine learning. *Nucleic Acids Res* 2023;51:D488–D508.
12. **Holm L, Laakso LM**. Dali server update. *Nucleic Acids Res* 2016;44:W351–W355.
13. **Guan L, Hariharan P**. X-ray crystallography reveals molecular recognition mechanism for sugar binding in a melibiose transporter MelB. *Commun Biol* 2021;4:1–13.
14. **Pettersen EF, Goddard TD, Huang CC, Meng EC, Couch GS, *et al.*** UCSF ChimeraX: Structure visualization for researchers, educators, and developers. *Protein Science* 2021;30:70–82.
15. **Severi E, Rudden M, Bell A, Palmer T, Juge N, *et al.*** Multiple evolutionary origins reflect the importance of sialic acid transporters in the colonization potential of bacterial pathogens and commensals. *Microb Genom* 2021;7:2021.03.01.433349.

16. **Severi E, Rudden M, Bell A, Palmer T, Juge N, *et al.*** Multiple evolutionary origins reflect the importance of sialic acid transporters in the colonization potential of bacterial pathogens and commensals. *Microb Genom* 2021;7:2021.03.01.433349.

17. **Robert X, Gouet P**. Deciphering key features in protein structures with the new ENDscript server. *Nucleic Acids Res* 2014;42:320–324.

18. **BLAKEY D, LEECH A, THOMAS GH, COUTTS G, FINDLAY K, *et al.*** Purification of the Escherichia coli ammonium transporter AmtB reveals a trimeric stoichiometry. *Biochemical Journal* 2002;364:527–535.
