# Supplementary figures and images for: Corrigendum: Characterisation of anhydro-sialic acid transporters from mucosa-associated bacteria
Source: Microbiology (Reading). 2024 Aug 2;170(8):001476. doi: 10.1099/mic.0.001476 (PMC11561582; doi:10.1099/mic.0.001476)

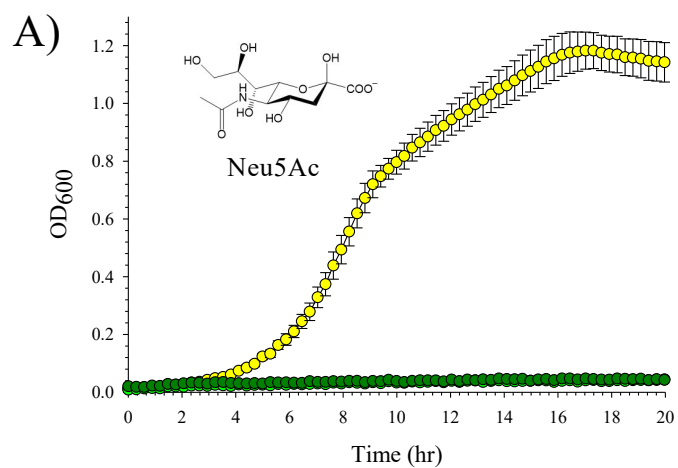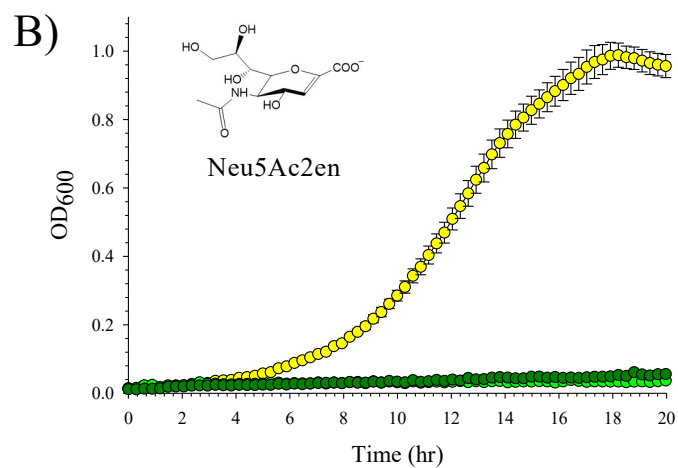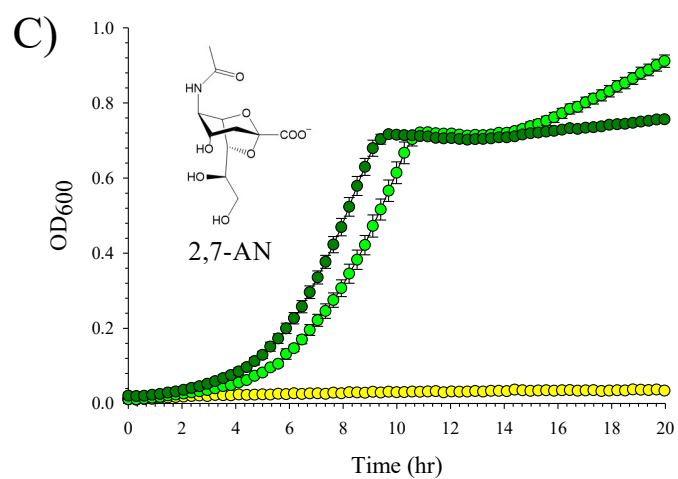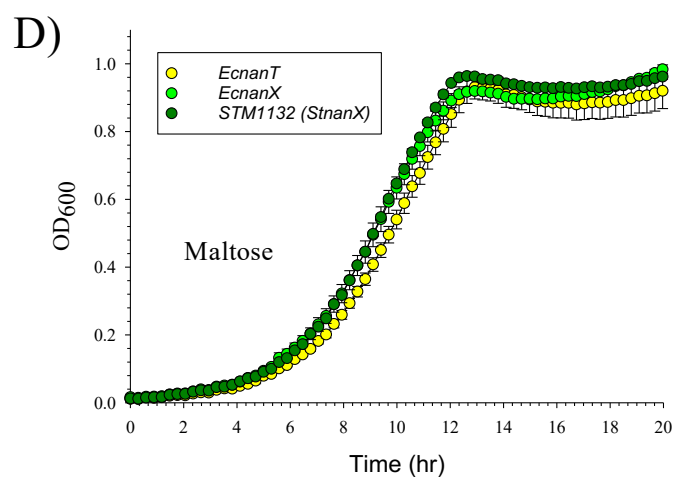

Supplement: Uncited Fig. S1. [file mic-170-01476-s001.pdf]
